# Epidemiology, Risk Factors and Seasonal Variation of Scrub Typhus Fever in Central Nepal

**DOI:** 10.3390/tropicalmed4010027

**Published:** 2019-02-02

**Authors:** Rajendra Gautam, Keshab Parajuli, Jeevan Bahadur Sherchand

**Affiliations:** Department of Microbiology, Maharajgunj Medical Campus, Institute of Medicine, Kathmandu 44600, Nepal; drkparajuli@iom.edu.np (K.P.); jeevanbsherchand@gmail.com (J.B.S.)

**Keywords:** scrub typhus, ELISA, *Orientia tsutsugamushi*, Nepal

## Abstract

Scrub typhus is a mite-borne acute febrile illness caused by *Orientia. tsutsugamushi,* a zoonotic bacterial infection common in the region known as the tsutsugamushi triangle. This study aims to determine the seroprevalence, seasonal variation, and risk factors of scrub typhus among the acute febrile illness patients attending different hospitals of central Nepal. Blood samples were collected from hospitalized patients of acute febrile illness suspected of scrub typhus infection attending different hospitals of central Nepal from April 2017 to March 2018. The IgM antibody to *Orientia tsutsugamushi* was detected by using the Scrub Typhus Detect™ Kit. Among the total cases (1585), 358 (22.58%) were positive for IgM Antibodies. Multivariate analysis identified several risks factors to be significantly associated with the scrub typhus infection, including gender (female) (odds ratio [OR] = 1.976, *p* ≤ 0.001, confidence interval [CI] = 1.417–2.756), rural residential location (odds ratio [OR] = 0.431, *p* = 0.001, confidence interval [CI] = 0.260–0.715), house near grassland (odds ratio [OR] = 3.288, *p* ≤ 0.001, confidence interval [CI] = 1.935–5.587), and working in the field (odds ratio [OR] = 9.764, *p* = 0.004, confidence interval [CI] = 2.059–46.315). The study findings indicate scrub typhus infection to be a significant health problem in Nepal. The proper diagnosis of infection cases, timely institution of therapy, public awareness, and vector control are important measures to be taken for the prevention and management of scrub typhus.

## 1. Introduction

Scrub typhus is a mite-borne acute febrile infectious illness caused by *Orientia tsutsugamushi*; a zoonotic bacterial infection common in the region known as the tsutsugamushi triangle which extends from northern Japan and far-eastern Russia in the north, to northern Australia in the south, and to Pakistan in the west [1]. Recent reports of scrub typhus caused by *Orientia* species other than *O. tsutsugamushi* well beyond the limits of the tsutsugamushi triangle have triggered concerns about the worldwide presence of scrub typhus [2]. The causative organism, *Orientia tsutsugamushi*, is transmitted to humans by the larval stage (chiggers) bite of the trombiculid mites, possibly *Leptotrombidium delience* [3]. Since these mites are widely distributed in different types of vegetation e.g., forests, rice paddies and plantations, farmers and people who engage in outdoor activities are at a higher risk of contracting scrub typhus [4]. Clinical manifestations are nonspecific, and they include acute febrile illness, fever, nausea, headache, shortness of breath, and myalgia. Recent studies on scrub typhus have reported the existence of various clinical manifestations with abnormal laboratory findings [5]. This disease is most common in resource limited settings such as rural areas and is difficult to differentiate clinically from other infections such as malaria, dengue, enteric fever and leptospirosis [6]. The aims of this study were to determine the seroprevalence, seasonal variation, risk factors, clinical characteristic and laboratory profile of scrub typhus among the acute febrile illness patients attending different hospitals of central Nepal.

## 2. Materials and Methods

A cross sectional descriptive study was conducted among hospitalized acute febrile illness patients with suspected scrub typhus cases in central Nepal for one year starting from April 2017 to March 2018. In brief, 1585 patients over the age of 1 year presenting with acute fever of more than 4 days were recruited into the study after excluding other obvious systemic or local causes of fever (such as respiratory tract infection, urinary tract infection, abscesses, cellulitis, etc) through clinical examination. Single Blood samples were collected from the hospitalized patients suspected of scrub typhus, presenting with acute febrile illness. The IgM antibody to *Orientia tsutsugamushi* was detected by using Scrub Typhus Detect™ Kit, In Bios International, USA, and the optical density was measured by HumaReader HS, ELISA reader, with optical density (OD) >0.50 being considered positive. The cut-off was calculated following recommendations for determining the endemic cut-off titre in the kit protocol. The cut-off calculated from a healthy volunteer was the mean OD (0.23) + 3 standard deviation (0.09) = 0.50. We proposed a cut-off OD value of >0.50 for Chitwan and the surrounding region based on our findings.

Written informed consent was obtained for each patient prior to their enrollment in the study. During the time of admission, a structured questionnaire was administered to assess the demographic variables of the patients who consented to the study. In addition, clinical characteristics and laboratory test results were recorded for the patients who were enrolled in the study. This study was approved by the Institutional Review Board of the Institute of Medicine, Tribhuvan University, Kathmandu, Nepal.

The collected data were entered in Epi info 3.5 from CDC and exported to IBM SPSS version 16.0 (SPSS Inc. Chicago, IL, USA). The association between the different demographic variables and the scrub typhus was determined using the chi square test, frequency distribution and univariate logistic regression analysis. Significant variables from the univariate logistic regression analysis were selected for the multivariate logistic regression analysis. An odds ratio with a 95% confidence interval was considered for the statistical significance. 

## 3. Results

### 3.1. Demographic Profile

Among the total scrub typhus cases (1585), 358 (22.58%) were positive for IgM antibodies (Table 1). A gender analysis of the infection cases revealed a female predominance (odds ratio [OR] = 1.976, *p* ≤ 0.001, confidence interval [CI] = 1.417–2.756) showing a significant association with scrub typhus. Students were most commonly affected (odds ratio [OR] = 2.231, *p* = 0.014, confidence interval [CI] = 1.174–4.238), followed by house wives (odds ratio [OR] = 2.054, *p* = 0.033, confidence interval [CI] = 1.060–3.982). Seropositivity was higher among the age group 51–60 years (28.5%), followed by the age group 1–10 years (22.7%) (Table 1).

### 3.2. Clinical Characteristics

Fever was the most common (100%) clinical characteristic observed in this study, followed by nausea (50.6%), headache (50.3%), shortness of breath (29.3%), abdominal pain (18.7%), tachypnea (16.8%), ventilation support (14.5%), jaundice (11.7%), hypertension (5.9%), seizure (4.7%), chronic pulmonary obstructive disease (COPD) (4.2%), diabetes (3.6%), hypotension(3.6%), and eschar (3.1%) (Table 2). 

### 3.3. Experimental Findings

Thrombocytopenia was the most common laboratory finding seen in 74.09% of the positive cases. A low level of hemoglobin was observed in 45.3% of patients. Leukocytosis was seen in 23.7% & leucopenia in 9.8%. A renal function test such as creatinine was raised in 18.7%, and a raised urea was observed in 13.4%. An assessment of the liver function showed the rise of liver enzymes, alanine amino transferases (ALT) was markedly increased in 77.4% cases, followed by aspartate amino transferase (AST) in 71.2%, alkaline phosphatase (ALP) in 32.1%, direct bilirubin in 32.1%, and total bilirubin in 24.3% (Table 3).

### 3.4. Antibiotics 

Doxycycline was the first drug choice for the treatment of scrub typhus in 43% of cases, followed by a combination of doxycycline, amoxicillin and azithromycin in 17.9% of cases. Doxycycline and azithromycin was used in 15.9% of cases, whereas ceftriaxone and azithromycin was used in 5.6% of cases. Table 4 describes the antibiotics used by attending physicians to treat 358 active cases of scrub typhus.

### 3.5. Seasonal Variation and Monthly Data

**Figure 1:**
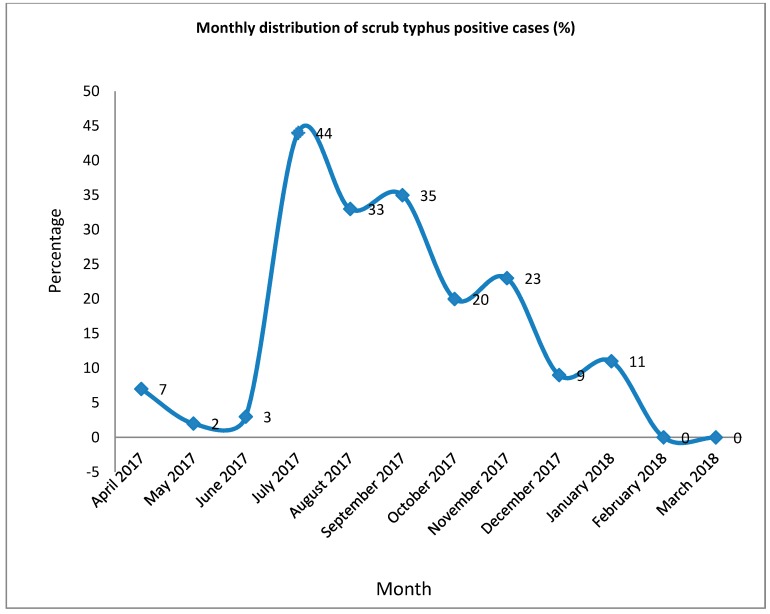
Monthly distribution of scrub typhus positive cases (percentage of total) indicating the highest percentage of positive cases in the month of July.

Scrub typhus infection was most commonly diagnosed during the month of July (43.6%), followed by September (35.4%) (Figure 1). An analysis of seasonal variation indicated that the summer (odds ratio [OR] = 17.879, *p* = 0.001, confidence interval [CI] = 5.303–60.276), fall/autumn (odds ratio [OR] = 25.141, *p* = 0.001, confidence interval [CI] = 7.528–83.970) and winter (odds ratio [OR] = 7.877, *p* = 0.004, confidence interval [CI] = 1.967–31.538) seasons were significantly associated with scrub typhus (Table 5).

### 3.6. Risk Factor Analysis

Most of the cases showing a significant association with scrub typhus infection were from rural areas (odds ratio [OR] = 0.431, *p* = 0.001, confidence interval [CI] = 0.260–0.715), patients residing near the grassland (odds ratio [OR] = 3.288, *p* ≤ 0.001, confidence interval [CI] = 1.935–5.587), patients with houses infested by mice (odds ratio [OR] = 5.504, *p* ≤ 0.001, confidence interval [CI] = 4.074–7.435) and people working in the field (odds ratio [OR] = 9.764, *p* = 0.004, confidence interval [CI] = 2.059–46.315) (Table 5).

## 4. Discussion

Scrub typhus is a common cause of acute febrile illness in Nepal. a multivariate analysis demonstrated that the following factors were significantly associated with the scrub typhus.

1. Females
2. Rural residential locations
3. Houses near grassland
4. The presence of mice inside the house and
5. Working in the field.

A total of 1585 patients suspected of having scrub typhus were included in the study to detect the presence of the IgM antibody by ELISA. The prevalence of scrub typhus was found to be at 22.58%, which is similar to the results of the studies conducted in Bangladesh and India [7,8]. A scrub typhus IgM ELISA was first developed in 1979, when it was shown to have a similar sensitivity and specificity to the IFA [9]. An assay utilizing the *O. tsutsugamushi*-specific recombinant 56-kDa antigen is now available as a commercial kit and more recent studies have demonstrated a similar performance, with sensitivities in the range of 85 to 93% and specificities between 94 and 97.5% [10,11,12]. The diagnostic cut-off value suggested in this study (ie., >0.50) is similar to that reported in the previous study from India [13]. The study from northern Thailand determined the OD cut-off value to be between 0.3–0.6, depending on the reference comparator and the cut–off threshold [14]. The recent study from Bangladesh determined the cut-off value to be between 0.75–1.25 [15]. Although the ELISA does not require specific training or equipment, the cost of the kit may still be too high for some laboratories and its availability is limited in some scrub typhus endemic countries [16].

During the last three years, the incidence of scrub typhus fever has been reported in different districts of Nepal, with the prevalence rate recorded to be as high as 40.3% [17]. In this study, the seroprevalence of scrub typhus was found to be higher among females than among males, which is in congruence with the previous data published from the same geographical region of Nepal, Rajasthan India and Korea [18,19,20]. Housewives were severely affected in our study. This might be due to active involvement of females in household work, and field work in our country. In contrast, the study in India found that prevalence rate for scrub typhus was higher among males (54.3%) than among female (45.7%) [21]. Scrub typhus was most common in age group 51–60 years. A higher prevalence of antibodies against *O. tsutsugamushi* has been observed in older people (>50 or 60 years) in several studies [20,22,23], and this probably reflects an increased opportunities for exposure over the course of their lifetime.

People residing in rural areas with their house near the grasslands and working in the field had higher odds of acquiring scrub typhus. The following were the significant risk factors associated with scrub typhus found in this study: female, fall/autumn, summer, presence of mice and working in the field. Seroprevalence and outbreak studies have identified various occupational and behavior risk factors, which include being a farmer (particularly on dry, cultivated land), working in vegetable fields, bundling waste straw, living at the edge of a village, sitting on grass whilst taking breaks and having close contact with rats, for the exposure to the causative organism *O. tsutsugamushi* in different countries [22,24,25]. Factors that may have resulted in increased risk were identified in some of the outbreaks, including patients squatting when relieving themselves in the bushes [26]. The primary hosts of the trombiculid mite larvae are wild rodents. Many studies have demonstrated the presence of *O. tsutsugamushi* in these animals [27], and the larval mites successfully transmit the bacterium to their rodent hosts [3]. Rodents are important in the ecology of scrub typhus and it is likely that infected rodents provide the explanation for the occurrence of multiple strains of *O. tsutsugamushi* within individual larvae [28]. Rodent populations in these rural areas were responsible for the transmission of the disease in human beings. Precautionary measures should be taken in the rural area to control the rodent population.

The seasonal variation of scrub typhus varies with the climate of the particular place. The transmission of disease occurs throughout the year in mountainous regions. In our study, the autumn, summer and winter seasons were in particular associated with the outbreak of scrub typhus cases. The scrub typhus outbreak starts after the monsoon and rainy season with the peak incidence in July followed by September. This might be because the occurrence of *Leptotrombidium deliense* is influenced by rainfall, with more chiggers attached to rodents during the wetter months of the year [29]. The Peak Incidence of scrub typhus outbreak in our neighboring country India was observed during the highest rainfall [30]. In Bangladesh, a strong seasonal pattern in incidence, with an increase in scrub typhus cases before and at the end of the rainy season and a decrease of cases in the middle of the dry season were observed [31]. In contrast no seasonal variation was observed in Taiwan [32]. 

Fever is the most common clinical characteristic present in scrub typhus patients which must be differentiated from other acute febrile illnesses [7,33]. Scrub typhus was the leading cause of acute undifferentiated fever in Chiangrai, northern Thailand (22.5%) and in Chittagong, Bangladesh (16.8%) [31,34]. The common clinical characteristics seen in this study were fever, headache, nausea, abdominal pain, tachypnea, shortness of breath and seizures. Our findings are in correlation with other studies conducted in Korea, Bangladesh, India, and Thailand [5,25,35,36,37].

Thrombocytopenia is the most common hematological parameter with leucocytosis and a low level of hemoglobin in scrub typhus patients. These finding are similar with studies conducted in India and Korea [38,39,40]. Elevated Transaminase with raised bilirubin and renal dysfunction were observed in our studies, and these were common laboratory abnormalities found in other studies [41,42,43,44].

The central pathophysiological derangements of thrombocytopenia, liver function and renal function in scrub typhus results from wide spread vasculitis and perivasculitis of these organs. This is due to the multiplication of the organism in the endothelial cells lining the small blood vessels and the consumption of platelets during the process of intravascular microthrombosis [45].

The limitation of this study includes the collection of single serum specimens for the detection of IgM antibodies during the acute phase of the disease. A rise in the antibody titre in paired sera could not be detected in our study.

## 5. Conclusions

Scrub typhus is an important public health problem in countries with tropical and mountainous regions like Nepal. This study found that at least 22% (one in every five) of acute febrile illnesses could likely be attributed to scrub typhus. Acute febrile illness patients must be investigated for scrub typhus along with other similar types of infections, and clinicians should be aware of this disease. The result of this study describes the demographic trend, risk factors, seasonal variation, clinical characteristics and laboratory findings in scrub typhus. The findings of this study may warrant the Ministry of Health, Nepal to recognize the burden of scrub typhus and intervene to reduce the threat. A proper confirmation of the diagnosis, early institution of therapy, public awareness and vector control are important factors to be taken into consideration in the prevention and management of scrub typhus.

## Figures and Tables

**Table 1:** Association of socio demographic variables and enzyme-linked immunosorbent assay (ELISA) report.

Variables	ELISA Report		*p*-Value	Odds Ratio	95% Confidence Interval
	Positive	Negative			
**Sex**					
Male	138 (16.7%)	690 (83.3%)		1	
Female	220 (29.1%)	537 (70.9%)	<0.001	0.488	0.384–0.621
**Occupation**					
Housewives	78(28.3%)	198(71.7%)	<0.001	3.008	1.889–5.051
Daily wages	17(26.2%)	48 (73.8%)	0.004	2.777	1.390–5.549
Students	171(24.9%)	517(75.1%)	<0.001	2.593	1.652–4.070
Farmers	67(20.0%)	268(80.0%)	0.008	1.960	1.195–3.215
Other [business, job]	25 (11.3%)	196(88.7%)		1	
**Education**					
Secondary level	74(24.3%)	231(75.7%)	0.125	2.323	0.791–6.823
Primary level	99(22.9%)	334(77.1%)	0.161	2.149	0.738–6.260
Higher secondary	85(22.4%)	295(77.6%)	0.178	2.089	0.714–6.108
No education	96(22.1%)	338(77.9%)	0.186	2.059	0.707–6.001
Graduate	4(12.1%)	29(87.9%)		1	
**Age Group**					
1–10 Years	61(22.7%)	208(77.3%)	0.046	2.236	1.015–4.929
11–20	80(26.3%)	224(73.7%)	0.012	2.723	1.248–5.940
21–30	62(21.5%)	226(78.5%)	0.067	2.092	0.951–4.603
31–40	53 (22.6%)	181(77.4%)	0.049	2.233	1.005–4.959
41–50	39(22.0%)	138(78.0%)	0.066	2.155	0.951–4.884
51–60	45 (28.5%)	113(71.5%)	0.007	3.037	1.345–6.853
61–70	10(11.6%)	76(88.4%)	0.995	1.003	0.373–2.697
71 and above	8 (11.6%)	61(88.4%)		1	
**Seasonal variation**					
Spring	3 (2.3%)	130(97.7%)		1	
Winter	10(7.5%)	124(92.5%)	0.062	3.495	0.940–12.996
Fall/Autumn	236(24.9%)	713(75.1%)	<0.001	14.343	4.523–45.480
Summer	109(29.5%)	260(70.5%)	<0.001	18.167	5.660–58.312
**Residential location**					
Rural	279 (23.8%)	892 (76.2%)	0.048	1.326	1.003–1.754
Urban	79 (19.1%)	335 (80.9%)		1	
**Type of house**					
Cemented floor	84 (20.0%)	336 (80.0%)		1	
No cemented floor	274 (23.5%)	891 (76.5%)	0.140	0.813	0.618–1.070
**House near grassland**					
Yes	303 (25.4%)	891 (74.6%)	<0.001	2.078	1.518–2.842
No	55 (14.1%)	336 (85.9%)		1	
**Piling weeds in house**					
Yes	63 (23.8%)	202 (76.2%)	0.613	1.084	0.794–1.479
No	295 (22.3%)	1025(77.7%)		1	
**Piling weeds on the yard**					
Yes	239(41.8%)	333(58.2%)	<0.001	5.392	4.185–6.947
No	119(11.7%)	894(88.3%)		1	
**Presence of mice**					
Yes	268(42.3%)	365(57.7%)	<0.001	7.032	5.377–9.197
No	90(9.5%)	862(90.5%)		1	
**Working in the field**					
Yes	237(43.8%)	304(56.2%)	<0.001	5.947	4.611–7.670
No	121 (11.6%)	923(88.4%)		1	

Note: piling weeds in house refers to the storage of grass inside the house which is mainly used for domestic animals.

**Table 2:** Clinical characteristics seen in scrub typhus cases (*n* = 358).

Clinical Characteristics	Number (%)
Fever	358 (100)
Nausea	181 (50.6)
Headache	180 (50.3)
Shortness of breath	105 (29.3)
Abdominal pain	67 (18.7)
Tachypnea	60 (16.8)
Ventilation support	52 (14.5)
Jaundice	42 (11.7)
Hypertension	21 (5.9)
Seizure	17 (4.7)
COPD	15 (4.2)
Diabetes	13 (3.6)
Hypotension	13 (3.6)
Eschar	11 (3.1)
Pregnancy	5 (1.4)

**Table 3:** Laboratory parameters of scrub typhus cases (*n* = 358).

Laboratory Parameters	Value	Number (%)
Hemoglobin	<11.0 gm/dL	163 (45.3)
	>11.0 gm/dL	196 (54.7)
Total leucocyte count	<4000 cumm	35 (9.8)
	4000–11,000 cumm	238 (66.5)
	>11,000 cumm	85 (23.7)
Platelet count	<150,000/µL	265 (74.09)
	150,000–450,000/µL	93 (26.0)
Urea	>45 mg/dL	48 (13.4)
	<45 mg/dL	310 (86.6)
Creatinine	>1.4 mg/dl	67 (18.7)
	<1.4 mg/dl	291 (81.3)
Bilirubin(total)	>1.2 mg/dL	87 (24.3)
	Up to 1.2 mg/dL	271 (75.7)
Bilirubin(direct)	>0.4 mg/dL	115 (32.1)
	Up to 0.4 mg/dL	243 (67.9)
AST	>45 mg/dL	255 (71.2)
	Up to 45 mg/dL	103 (28.8)
ALT	>40 mg/dL	277 (77.4)
	Up to 40 mg/dL	81 (22.6)
ALP	>192.0 U/L	115 (32.1)
	<192.0 U/L	243 (67.9)
Protein	>6.0 mg/dL	276 (77.1)
	Up to 6.0 mg/dL	82 (22.9)
Albumin	>3.5 mg/dL	119 (33.2)
	Up to 3.5 mg/dL	239 (66.8)

**Table 4:** Antibiotics prescribed in scrub typhus cases (*n* = 358).

	Number (%)
Doxycycline	154 (43.0)
Doxycycline/Ciprofloxacin/Azithromycin	64 (17.9)
Doxycycline/Azithromycin	57 (15.9)
Ceftriaxone/Azithromycin	20 (5.6)
Azithromycin	18 (5.0)
Piperacillin/Tazobactum	16 (4.5)
Amikacin IV	13 (3.6)
Levofloxacin/Ceftazidime	9 (2.5)
Ceftriaxone IV	7 (2.0)

**Table 5:** Multivariate analysis of association of ELISA report with socio demographic variables.

Variables	ELISA Report		*p*-Value	Adjusted Odds Ratio	95% Confidence Interval
	Positive	Negative			
**Sex**					
Male	138 (16.7%)	690 (83.3%)		1	
Female	220 (29.1%)	537 (70.9%)	<0.001	1.976	1.417–2.756
**Occupation**					
Housewives	83(28.3%)	210(71.7%)	0.033	2.054	1.060–3.982
Daily wages	16(25.8%)	46(74.2%)	0.092	2.115	0.886–5.049
Students	173(24.6%)	531(75.4%)	0.014	2.231	1.174–4.238
Farmers	66(19.2%)	277(80.8%)	0.105	1.704	0.895–3.245
Other [business, job]	20(10.9%)	163(89.1%)		1	
**Age groups**					
1–10 Years	61(22.7%)	208(77.3%)	0.171	2.042	0.736–5.667
11–20	80(26.3%)	224(73.7%)	0.329	1.690	0.589–4.848
21–30	62(21.5%)	226(78.5%)	0.381	1.557	0.578–4.191
31–40	53 (22.6%)	181(77.4%)	0.406	1.504	0.575–3.934
41–50	39(22.0%)	138(78.0%)	0.387	1.537	0.580–4.075
51–60	45 (28.5%)	113(71.5%)	0.077	2.403	0.908–6.357
61–70	10(11.6%)	76(88.4%)	0.665	0.773	0.240–2.485
71 and Above	8(11.6%)	61(88.4%)		1	
**Season**					
Spring	3 (2.3%)	130(97.7%)		1	
Winter	10(7.5%)	124(92.5%)	0.004	7.877	1.967–31.538
Fall autumn	236(24.9%)	713(75.1%)	<0.001	25.141	7.528–83.970
Summer	109(29.5%)	260(70.5%)	<0.001	17.879	5.303–60.276
**Residential location**					
Rural	279 (23.8%)	892 (76.2%)	0.001	1.32	1.245–1.675
Urban	79 (19.1%)	335 (80.9%)		1	
**House near grassland**					
Yes	303 (25.4%)	891 (74.6%)	<0.001	3.288	1.935–5.587
No	55 (14.1%)	336 (85.9%)		1	
**Piling weeds in yard**					
Yes	239(41.8%)	333(58.2%)	0.409	0.517	0.108–2.480
No	119(11.7%)	894(88.3%)		1	
**Presence of mice**					
Yes	268(42.3%)	365(57.7%)	<0.001	5.504	4.074–7.435
No	90(9.5%)	862(90.5%)		1	
**Working in the field**					
Yes	237(43.8%)	304(56.2%)	0.004	9.764	2.059–46.315
No	121 (11.6%)	923(88.4%)		1	
